# Changes in glucose metabolism in essential tremor: within and beyond the cerebello-thalamo-cortical circuit

**DOI:** 10.1093/braincomms/fcaf227

**Published:** 2025-06-18

**Authors:** Sarah Jonker, Jelle R Dalenberg, Debora E Peretti, A M Madelein van der Stouwe, Marina A J Tijssen

**Affiliations:** Expertise Centre Movement Disorders Groningen, University of Groningen, University Medical Center Groningen, Groningen 9700 RB, The Netherlands; Expertise Centre Movement Disorders Groningen, University of Groningen, University Medical Center Groningen, Groningen 9700 RB, The Netherlands; Laboratory of Neuroimaging and Innovative Molecular Tracers (NIMTlab), Geneva University Neurocenter and Faculty of Medicine, University of Geneva, Geneva CH-1211, Switzerland; Expertise Centre Movement Disorders Groningen, University of Groningen, University Medical Center Groningen, Groningen 9700 RB, The Netherlands; Expertise Centre Movement Disorders Groningen, University of Groningen, University Medical Center Groningen, Groningen 9700 RB, The Netherlands

**Keywords:** essential tremor, FDG-PET, cerebellum, cerebello-thalamo-cortical circuit

## Abstract

The pathophysiology and cerebellar role in essential tremor are not yet fully understood. Previous fludeoxyglucose positron emission tomography (FDG-PET) studies comparing glucose metabolism between essential tremor and healthy participants have led to few and inconsistent results. We aimed to examine changes in brain glucose metabolism in [^18^F]FDG PET brain imaging in 19 right-handed essential tremor patients and compare them to 19 right-handed healthy participants using a voxel-based mass univariate analysis. The Montreal Cognitive Assessment and the Hospital Depression scale were used as covariates. In addition, the correlation between tremor severity as measured with the Fahn-Tolosa-Marin Tremor Rating Scale and brain glucose metabolism in essential tremor patients was assessed. Essential tremor patients showed significantly higher metabolism in the right dentate nucleus and, at a more liberal threshold, lower metabolism in the right parietal cortex. A positive correlation was detected between glucose metabolism in the left paracentral region and tremor severity, again at more liberal thresholding. Beyond the cerebello-thalamo-cortical circuit, the decreased activity in the right parietal areas in essential tremor patients may indicate that the sensorimotor integration is an important aspect of essential tremor pathophysiology. In conclusion, our findings show altered glucose metabolism within and beyond the cerebello-thalamo-cortical circuit.

## Teaser

Essential tremor is currently considered a network disorder rather than an isolated cerebellar dysfunction. Sarah Jonker *et al*. studied essential tremor patients and healthy participants using [^18^F]fludeoxyglucose positron emission tomography (FDG-PET). The changes in brain metabolism further support the cerebello-thalamo-cortical circuit, but point also towards altered sensorimotor integration.

## Graphical Abstract

**Graphical Abstract:**
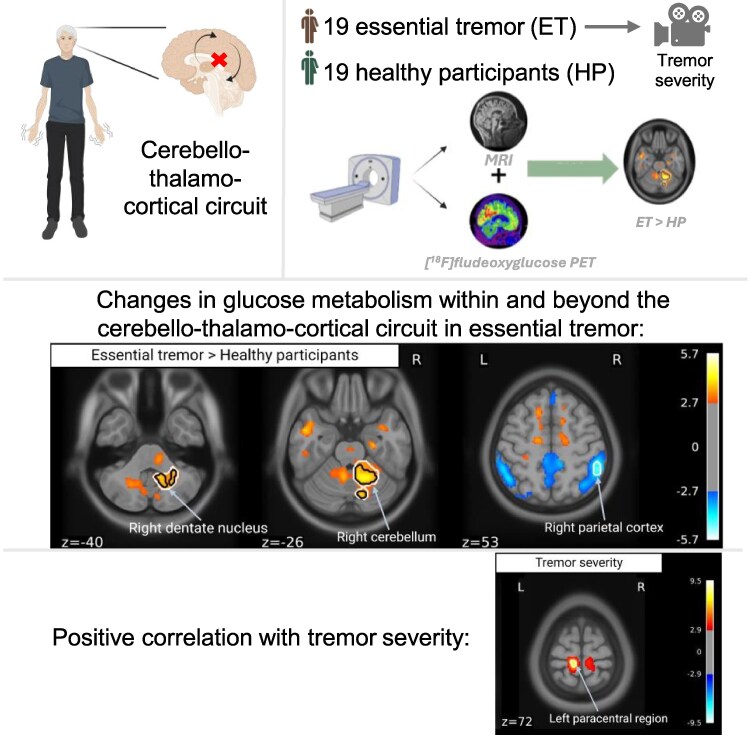

## Introduction

Essential tremor is the most common movement disorder worldwide, with a prevalence of 1.3% in the general population and 6% in the population older than 65 years.^1^ Essential tremor is a clinical diagnosis, which can be supported by clinical neurophysiological testing.^2,3^ Patients are affected by a postural and kinetic tremor of the upper limbs, and sometimes also of the head and voice. Disease onset is typically gradual; slowly progressing over decades. In more advanced disease stages, patients can experience difficulties with many activities of daily life, including writing, handling cutlery and using a computer or mobile phone. Several types of medication are available, however, if essential tremor proves medication-refractory, the ventral intermediate nucleus of the thalamus is the most targeted structure for neurosurgical intervention, whether by means of thalamotomy, deep brain stimulation, or more recently MR guided focused ultrasound.^4,5^

In contrast to clinical practice, where neuroimaging tests are only performed if an alternative diagnosis is under consideration,^6^ neuroimaging studies have proved their value in unravelling the pathophysiology of essential tremor.

Structural, functional, and metabolic neuroimaging studies have led to robust evidence that essential tremor results from pathological oscillations within the cerebello-thalamo-cortical circuit.^7^ Where these pathological oscillations arise is a matter of debate. They could originate directly from the cerebellum, in which case the cerebellum is seen as a driving oscillator (cerebellar oscillator hypothesis). Or essential tremor may be caused by cerebellar dysfunction, where the cerebellum is disconnected from the cerebello-thalamo-cortical circuit (cerebellar decoupling hypothesis). The cerebellar oscillator hypothesis is supported by previous studies reporting increased activity of the cerebellar cortex in essential tremor patients compared to healthy participants,^8-11^ which in a recent elegant study was shown to normalize when thalamic deep brain stimulation was turned on.^12^ Moreover, a recent translational study showed excessive cerebellar oscillations in both mouse models and essential tremor patients by means of cerebellar EEG.^13^ The cerebellar decoupling hypothesis, on the other hand, is supported by functional connectivity studies, where altered functional connectivity in the cerebello-thalamo-cortical circuit is consistently reported,^14,15^ while decreased dentate nucleus functional connectivity with cortical and cerebellar cortices was found to correlate with tremor amplitude.^16^

There have been relatively few fludeoxyglucose positron emission tomography (FDG-PET) studies in essential tremor and previous studies comparing brain glucose metabolism between essential tremor and healthy participants have led to few and inconsistent results. Three studies did not find changes in cerebellar metabolism,^17,18^ whereas one study found cerebellar hypometabolism upon treatment with propranolol.^19^ These previous studies were often done with relatively small sample sizes and did not take into account the participants’ handedness, nor adjust for any variability in cognitive performance or emotional states.^17-19^

To further increase our understanding of the brain pathophysiology in essential tremor and the role of the cerebellum therein, we performed an FDG-PET study in a cohort of essential tremor patients to study their brain glucose metabolism. We hypothesized that further research, in addition to the limited previous FDG-PET studies on essential tremor, will provide more insight into the underlying mechanism in essential tremor while adjusting for variability in cognitive performance and emotional state.

## Material and methods

The current study is a cross-sectional study in which we compare brain metabolism measured with [^18^F]FDG PET between 19 right-handed essential tremor patients and 19 healthy participants. For comparison, all individuals also receive an anatomical MRI scan. This study is part of the larger Next Move in Movement Disorders (NEMO) study.^20,21^

### Participants

Twenty-one patients participated in the study of which 19 were included in the analysis. Reasons for exclusion were left-handedness (*n* = 1) and ambidexterity (*n* = 1). Patients and healthy participants were matched based on age, and only right-handed individuals were included. Patients with a clinical diagnosis of essential tremor were recruited in the Netherlands from movement disorder clinics at the University Medical Center Groningen (UMCG), The Isala hospital Zwolle, The Medisch Spectrum Twente, and online via Hersenonderzoek.nl and essentieletremor.nl. Healthy participants were recruited through advertisements in the UMCG or were acquaintances of researchers involved in the NEMO study. The recruitment period spanned from July 2019 to December 2023.

Participants inclusion criteria for the current study were 16 years or older and right-handed. Exclusion criteria were: (i) other neurological conditions that lead to movement problems other than the hyperkinetic movement disorder, (ii) other conditions that lead to impaired hand or arm function, (iii) participants with a pacemaker, and (iv) contraindications for MRI. Moreover, healthy participants that were first-degree relatives of patients with movement disorders were additionally excluded. This study was approved by the medical ethical committee of the UMCG (METc 2018/444). Written informed consent was obtained from all subjects according to the Declaration of Helsinki.

### Clinical evaluation

For all essential tremor patients, the movement disorder phenotype was confirmed by three international hyperkinetic movement disorder experts. As described in the NEMO protocol, data are only used from patients when at least two out of three blinded movement disorders experts agreed on tremor as the dominant phenotype, with a Fleiss kappa across the patient group of >0.8 indicating excellent inter-rater agreement, based on offline assessment of videos and clinical information including clinical neurophysiology and neuroimaging test results if available. To assess cognitive performance, all participants filled out the Montreal Cognitive Assessment (MoCA).^22^ The tremor severity was assessed using the Fahn-Tolosa-Marin Tremor Rating Scale (FTM-TRS) part A.^23^ This was done offline by video assessment of rest, posture, and intentional movement of both arms as well as head tremor by one independent movement disorders neurologist (Dr. Giorgia Sciacca, see acknowledgements). Furthermore, anxiety and depression were assessed using the Hospital Anxiety and Depression scale (HADS).^24^

### Procedure

Subjects were instructed to avoid any physical activity and to fast for a minimum of 6 h prior to scanning. Plasma glucose levels were evaluated to ensure adherence to this fasting procedure. Scanning was only performed in subjects with plasma glucose levels <11 mmol/L.

Subjects that met this criterion received a bolus of an average of 199.25 MBq (± 0.09) [18F]FDG, provided via an intravenous catheter. Subsequently, the canulla was flushed out with 10 mL buffered saline. In the 30 min period between the FDG injection and radiotracer image acquisition, subjects rested in a supine position, while awake in a quiet room with the lights dimmed.

### Data acquisition

The data acquisition protocol for the NEMO study protocol has been published previously.^21^ In summary, at the Nuclear Medicine and Molecular Imaging department of the University Medical Centre Groningen, PET images were obtained by either a 64mCT PET/CT or Siemens Biograph 40 scanner. Dynamic acquisition occurred over 10 min (5 sessions of 2 min), merging images into a single static scan. 3D OSEM, point spread function and time-of-flight corrections were used to generate 2 mm isotropic voxels in a 400 × 400 × 111 matrix, with a 2 mm Gaussian filter for smoothing. Radiotracers were produced per Good Manufacturing Practices at UMCG. MRI was performed with a 3T Siemens Prisma scanner and a 64-channel head coil, utilizing T1-weighted parameters: TE: 2.98 ms; TR: 2300 ms; FOV: 256; FA: 9°; and 176 sagittal slices (1 mm thickness). Subjects were instructed to fixate on a cross. For essential tremor subjects with head tremors, a dynamic acquisition protocol collected 5 images every 2 min. A frame-based registration approach corrected motion, merging the images for analysis without using restrictive devices to ensure comfort.

### Image processing

A robust in-house pipeline was utilized to pre-process the images. We started with pre-processing the anatomical images in fMRIPrep (version 20.2.0) resulting in anatomical images (bias corrected) and standardization of the parameters for conversion of the images to MNI space (*MNI152NLin2009cAsym* with 2 mm resolution). PET images were then pre-processed using Nipype (v1.8.3), starting with using AFNI’s autobox function (v21.3.04) to crop PET images followed by applying HD-BET (v1.0) for brain extraction. In two stages, Normalization Tools (ANTs, v2.3.512) was used to co-register brain extracted images to bias corrected anatomical images; first, the brain mask was co-registered to the anatomical image, second, the brain extracted PET image was used for a more detailed co-registration process.

Linear co-registration transformations were merged with the normalization transformation matrix from fMRIprep and applied to the original PET image.

Subsequently, a voxel-based analysis was performed using Nilearn 0.10.2^25^ running in Python 3.11.1. Since we could not define a robust reference area to calculate standardized uptake value ratios for these patients, we first scaled data through z-scoring all voxels within the brain mask of the MNI template; for each participant, the mean of all voxels was subtracted and the values were divided by the standard deviation these voxels: (X - μ)/SD.^26-28^ As a result, uptake values represent the relative uptake compared to all other voxels in the brain. A general linear model framework was used to test for differences between the experimental groups.

### Statistical analysis

Power analyses for the number of participants are very uncommon for PET studies. Consequently, we elected sample sizes for patients with hyperkinetic movement disorders that have been frequently documented in the literature. The most recent systematic review by Timmers^29^ analysed 104 [^18^F]FDG PET studies performed in patients with different hyperkinetic movement disorders. The number of patients in the included studies ranged between 5 and 50, and the average was 15.2. For the studies that involved tremor the average was 17.

Analyses of demographic and clinical characteristics were performed in R 4.3.1 (R Core Team, 2023). Two sample *t*-tests were used to test for group differences in demographic and clinical characteristics.

For the voxel-based PET analysis, we smoothed images with a 10 mm FWHM kernel and performed mass univariate regression, using group as independent variable, and MoCA, depression and tremor severity scores were evaluated as covariates. To assess any possible effects of rest tremor during the scans, we summed rest tremor scores from the FTM-TRS as a separate covariate.

In the text, we will report exploratory results at a liberal *P* < 0.1 corrected threshold and at a significant *P* < 0.05 corrected threshold using threshold-free cluster enhancement (TFCE) permutation statistics (1000 permutations).^30^ To enhance visual interpretation of our results, we chose to lower thresholding for visualizing results as per recommendation by Taylor *et al*.^31^ Thresholds for figures are uncorrected *P* < 0.01, with additional white and black contours delineating results surviving thresholds of *P*_(TFCE)_ < 0.1 and *P*_(TFCE)_ < 0.05. While this means there will be results visible subthreshold, this has the advantage for the reader to see the results in a larger spatial context: the purpose is not to draw statistical conclusions, but rather to facilitate interpretation in the context of activation patterns. For tables, we chose to include fMRI results surviving a threshold of *P*_(TFCE)_ < 0.1, corresponding to the white contours in the figures and the threshold for reporting results in the text.

## Results

### Clinical characteristics

Table 1 shows an overview of the clinical characteristics of the 19 right-handed essential tremor patients and the 19 right-handed healthy participants that were included in the study. Inter-rater agreement between movement disorders experts on tremor as the primary phenotype was unanimous (3/3) for all patients. No significant differences between groups were found in the covariates. We chose not to include HADS anxiety scores because of high collinearity between the HADS depression and anxiety scores (Pearson correlation of 0.86, *P* < 0.001). As we also found a high correlation between MoCA and age (*r* = −0.45, *P* = 0.005), we chose to only include MoCA as a covariate as a reflection of cognitive well-being. We found a resting tremor in 11 patients (11/19 of ET patients, 8/11 unilaterally, 3/11 bilaterally, median summed FTM-TRS score for rest tremor of 1, range 0–5). In total, there were 5 out of 38 missing values at random in each of the three covariates, 2 in the essential tremor group and 3 in the healthy participant group. These missing values were imputed using the mean of the respective group.

**Table 1:** Clinical characteristic of the healthy participants and essential tremor patients

	HP (*n* = 19)	ET (*n* = 19)	Statistic
Mean age (SD)	58.79 (17.57)	64.63 (14.45)	Welch *t*(34.71)=−1.12, *P* = 0.27
Sex	7F/12M	5F/14M	χ^2^(1) = 0.12, *P* = 0.73
Mean age at onset (SD, range)^a^		38 (24.84, 1–70)	
Mean tremor severity (FTM-TRS A) (SD)		7.16 (4.17)	
Mean MoCA (SD)^a^	26.38 (2.85)	27 (1.58)	Welch *t*(23.14)=−0.78, *P* = 0.45
Mean anxiety (SD)^a^	2.81 (2.59)	2.94 (3.05)	Welch *t*(30.68)=−0.13, *P* = 0.90
Mean depression (SD)^a^	1.94 (2.26)	3.06 (3.54)	Welch *t*(27.2)=−1.10, *P* = 0.28

HP, healthy participants; F, female; M, male; ET, essential tremor; FTM-TRS, Fahn-Tolosa-Marin Tremor Rating Scale part A.

^a^Five missing values at random.

### Metabolic differences between healthy participants and essential tremor patients

**Figure 1:**
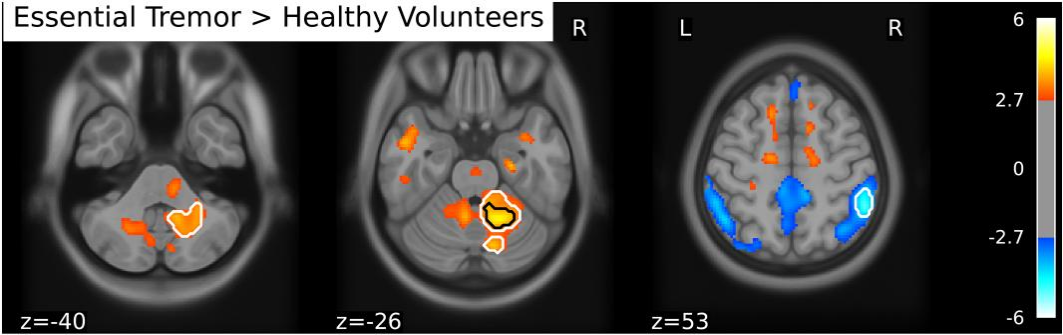
**Metabolic differences between essential tremor patients and healthy participants.** Mass univariate regression with group as independent variable. Contrast colouring shows higher brain metabolism for essential tremor patients (*n* = 19) in red, whereas higher brain metabolism for healthy participants (*n* = 19) is shown in blue. Colour coding represents *t*-values that are thresholded at *P* < 0.01 uncorrected for enhanced interpretability. Voxels surviving a liberal statistical thresholding of *P*_(TFCE)_ < 0.1 are depicted with white contours. Voxels surviving statistical thresholding of *P*_(TFCE)_ < 0.05 are depicted with black contours.

**Figure 2:**
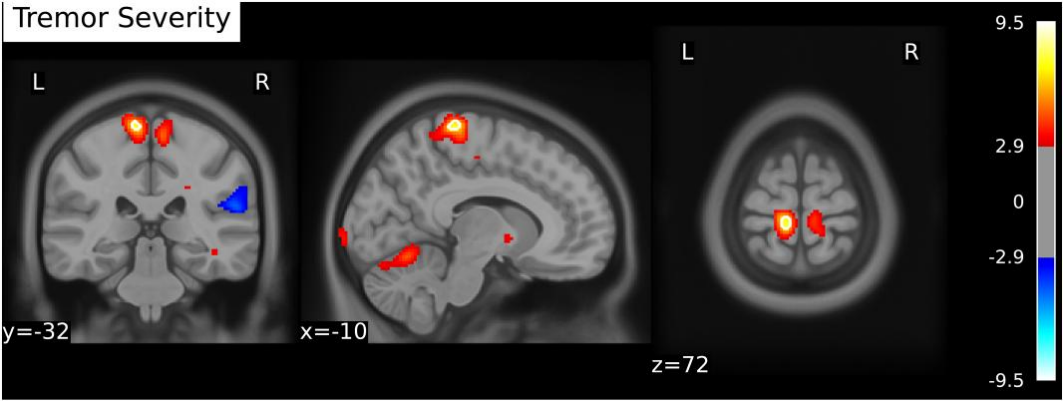
Brain regions correlating with tremor severity in essential tremor patients. Mass univariate regression in essential tremor patients (*n* = 19) with tremor severity (FTM TRS) (A) as covariate. Contrast colouring shows increased tremor severity in red, whereas decreased tremor severity is shown in blue. Colour coding represents *t*-values that are thresholded at *P* < 0.01 uncorrected for enhanced interpretability. Voxels surviving statistical thresholding of *P*_(TFCE)_ < 0.1 are depicted with white contours.

To investigate differences in brain metabolism between essential tremor patients and healthy participants, we tested the contrast [essential tremor > healthy participants] while using MoCA and HADS depression scores as covariates. We also considered resting tremor as covariate. However, rest tremor did not substantially change the results. Given that resting tremor would consume an additional degree of freedom without contributing meaningful explanatory power, we excluded it from the final model. We found that brain metabolism in the right dentate nucleus was significantly higher in essential tremor patients than in healthy participants (*t*(33) = 4.45, *P*_(TFCE)_ = 0.025). At a liberal threshold, we also found lower metabolism in essential tremor patients compared to healthy participants in right parietal regions (*t*(33) = −4.91, *P*_(TFCE)_ = 0.067). Furthermore, we detected a positive relation between tremor severity and glucose metabolism in the left paracentral lobe at liberal thresholding (*t*(15) = 7.48, *P*_(TFCE)_= 0.082.). Results are shown in Tables 2 and 3; Figs. 1 and 2. For reference and future meta-analyses, group differences at a conventional uncorrected threshold of *P* < 0.001 are available in Supplementary Table 1.

**Table 2:** Essential tremor versus healthy participants peak statistics

Area	MNI coordinates			Uncorrected statistic			Corrected statistic (TFCE)	
	*X*	*Y*	*Z*	*T*	*P*	Size (mm^3^)	*P*	Size (mm^3^)
Right cerebellum	16	−54	−26	4.45	<0.001	3824	0.025	5224^a^
Right dentate nucleus	26	−56	−36	4.26	<0.001		0.026	
Right cerebellum	12	−54	−38	4.07	<0.001		0.036	
Right cerebellum	12	−72	−28	3.92	<0.001	232	0.049	424^a^
Right inferior parietal lobe	52	−44	54	−4.91	<0.001	2064	0.067	568^b^

^a^Thresholded at *P*_(TFCE)_ < 0.05.

^b^Thresholded at *P*_(TFCE)_ < 0.1.

**Table 3:** Correlation tremor severity and metabolism

ID	Area	MNI coordinates			Uncorrected statistic			Corrected statistic (TFCE)	
		*X*	*Y*	*Z*	*T*	*P*	Size (mm^3^)	*P*	Size (mm^3^)
1	Left paracentral lobule	−10	−32	72	7.48	<0.001	400	0.082	96^a^

^a^Thresholded at *P*_(TFCE)_ < 0.1.

## Discussion

In this study, we aimed to investigate brain glucose metabolism in essential tremor patients and healthy participants using [^18^F]FDG PET brain imaging and detected differences in various brain regions encompassing areas both within and beyond the cerebello-thalamo-cortical circuit. Moreover, tremor severity correlated positively with increased glucose metabolism in the left paracentral lobule.

In brief, the cerebello-thalamo-cortical circuit involved in essential tremor comprises the cerebellar cortex, the dentate nucleus, the thalamus, and the motor, premotor, and supplementary motor area of the cortex. Within this network, we found significantly increased glucose metabolism in the right cerebellum of essential tremor patients. This has not been demonstrated before in [^18^F]FDG PET studies,^17,18^ yet fits well with results of increased cerebellar activity,^8,9^ or increased cerebellar blood flow from previous functional MRI (fMRI) and regional blood flow (rCBF) PET studies.^10,11,32^ The absence of cerebellar metabolism changes in previous [^18^F]FDG PET studies might be because in these studies the participants’ handedness was not taken into account, whereas here we included only right-handed patients and healthy participants.^17,18^ As handedness affects motor organization in the brain,^33-35^ we decided to only include right-handed participants to maximize homogeneity regarding lateralized neuronal organization of motor control, which is customary in task-based fMRI studies on essential tremor.^8,36-38^ This factor may have increased our power to detect changes in the cerebellum. Moreover, in previous [^18^F]FDG PET studies no analyses were set up to account for variability in cognitive performance and mood. Our novel finding of increased cerebellar metabolism fits the cerebellar oscillator hypothesis of essential tremor pathophysiology, in which pathological oscillations are hypothesized to originate directly from the cerebellum, which then acts as the driving oscillator.

Regarding the thalamus, we did not find any changes in glucose metabolism, even at the more liberal uncorrected threshold, thus providing very little evidence for robust group differences within this rain region during rest. However, results from previous studies investigating glucose metabolism in essential tremor have been mixed: two studies reported either increased or decreased thalamic metabolism,^17,39^ while two other studies report no thalamic changes at all.^18,19^ Despite what is currently known about the role of the thalamus (and VIM especially) in essential tremor pathophysiology, changes in glucose metabolism of the thalamus at rest appear not to be a consistent hallmark of essential tremor.

Beyond the cerebello-thalamo-cortical circuit, changes in essential tremor patients also consisted of decreased brain glucose metabolism in the right parietal cortex, at the more liberal threshold of *P* < 0.1, TFCE-corrected. Specifically, decreased metabolism was observed in the inferior parietal lobule in essential tremor patients compared to healthy participants, an area concerned with sensory processing and sensorimotor integration. This finding fits with two consecutive Korean studies, where reduced glucose metabolism was reported in the precuneus of the right parietal lobe in essential tremor patients compared to healthy participants.^18,19^ Similarly, reduced activity in the right parietal cortex has also been reported before in essential patients performing a rhythmic finger tapping task in an fMRI experiment.^36^ Moreover, in a machine learning MRI study investigating cortical thickness, roughness (i.e. the standard deviation of cortical thickness as a measure for thinning) of the right inferior parietal area was shown to be a key feature in distinguishing essential tremor patients from healthy participants.^40^ Finally, in a previous fMRI study involving a grip-force task, it was shown that increasing visual feedback led to exacerbation of essential tremor and this increase was associated with changes in BOLD amplitude in the inferior parietal lobule, as well as within the cerebello-thalamo-cortical circuit.^38^ Together, our findings further support that the inferior parietal lobule is involved in essential tremor, which may indicate that sensorimotor integration is an important aspect of essential tremor pathophysiology.

Interestingly, we detected a positive correlation between tremor severity and increased glucose metabolism in the left paracentral lobule. This correlation was established at the more liberal threshold of *P* < 0.1 TFCE-corrected, and indicates that the more severe a patient’s tremor, the higher the glucose metabolism. The left paracentral lobule contains a part of the supplementary motor area in its anterior portion and the somatosensory cortex in its posterior portion.^41^ Similar correlation between tremor severity and structural changes in the paracentral lobule has been described in a previous MRI study. However, this correlation involved the right instead of the left paracentral lobule, possibly due to the inclusion of both left- and right-handed participants.^42^ Moreover, two previous resting state fMRI studies have shown increased paracentral lobule activity in essential tremor patients compared to healthy participants.^43,44^ Our new finding of a positive correlation between tremor severity and metabolism in the paracentral lobule further supports involvement in essential tremor of this area, located firmly within the cerebello-thalamo-cortical circuit.

A few methodological considerations need to be addressed. A limitation relates to the nature of [^18^F]FDG PET studies, in which participants are required to lie as still as possible, and essential tremor, which is a movement disorder of action. This ‘mismatch’ can lead to two potential problems. First, because patients have no or minimal tremor during recording, functional brain changes are more difficult to detect rendering the method unsuitable, however, we were able to establish clear differences between essential tremor patients and healthy participants. Second, some essential tremor patients experience trembling of the head as well as the hands, which can lead to movement artefacts. We mitigated this issue as best we could by choosing a dynamic acquisition protocol.

In conclusion, our findings show altered glucose metabolism in the cerebello-thalamo-cortical circuit, partly correlating with tremor severity, as well as parietal areas in essential tremor patients compared to healthy participants. These functional changes further support that essential tremor is a network disorder.

## Supplementary Material

fcaf227_Supplementary_Data

## Data Availability

The data that support the findings of this study are available from the corresponding author, upon reasonable request. The pre-processing pipeline is available at https://github.com/jrdalenberg/PETBrainPreprocessing.
