# Supplementary material for: Changes in glucose metabolism in essential tremor: within and beyond the cerebello-thalamo-cortical circuit
Source: Brain Commun. 2025 Jun 18;7(3):fcaf227. doi: 10.1093/braincomms/fcaf227 (PMC12188438; doi:10.1093/braincomms/fcaf227)
Supplement: fcaf227_Supplementary_Data [file fcaf227_supplementary_data.docx]

**Supplementary Table 1. Overview of brain regions in essential tremor, both TFCE corrected and uncorrected.**

| ID | Area | MNI Coordinates | | | Uncorrected Statistic | | | Corrected Statistic (TFCE) | |
| --- | --- | --- | --- | --- | --- | --- | --- | --- | --- |
|  |  | X | Y | Z | T | p | Size  (mm3) | p | Size  (mm3) |
| 1 | Left middle temporal gyrus | -54 | -22 | -12 | 5.72 | <0.001 | 1200 | n.s. |  |
| 2 | Right cerebellum | 16 | -54 | -26 | 4.45 | <0.001 | 3824 | <0.05 | 5224 |
| 3a  3b | Right dentate nucleus  Right cerebellum | 26  12 | -56  -54 | -36  -38 | 4.26  4.07 | <0.001  <0.001 |  | <0.05  <0.05 |  |
| 4 | Left middle frontal lobe | -26 | 10 | 38 | 4.12 | <0.001 | 328 | n.s. |  |
| 5 | Right parahippocampal gyrus | 26 | -18 | -24 | 4.05 | <0.001 | 64 | n.s. |  |
| 6 | Right insula | 38 | -8 | -12 | 3.95 | <0.001 | 272 | n.s. |  |
| 7 | Left middle temporal gyrus | -44 | 8 | -30 | 3.94 | <0.001 | 280 | n.s. |  |
| 8 | Left postcentral gyrus | -52 | -14 | 28 | 3.93 | <0.001 | 248 | n.s. |  |
| 9 | Left SMA | -12 | 4 | 60 | 3.92 | <0.001 | 136 | n.s. |  |
| 10 | Right cerebellum | 12 | -72 | -28 | 3.92 | <0.001 | 232 | <0.05 | 424 |
| 11 | Fusiform gyrus | -46 | -62 | -20 | 3.89 | <0.001 | 144 | n.s. |  |
| 12 | Right insula | 34 | 12 | -4 | 3.87 | <0.001 | 56 | n.s. |  |
| 13 | Left precentral gyrus | -40 | -10 | 44 | 3.84 | <0.001 | 64 | n.s. |  |
| 14 | Left putamen | -30 | -10 | -4 | 3.84 | <0.001 | 160 | n.s. |  |
| 15 | Left superior frontal gyrus | -16 | 54 | 24 | 3.80 | <0.001 | 48 | n.s. |  |
| 16 | Left cerebellum | -22 | -62 | -36 | 3.79 | <0.001 | 88 | n.s. |  |
| 17 | Right middle cingulate cortex | 16 | 4 | 38 | 3.79 | <0.001 | 200 | n.s. |  |
| 18 | Right parahippocampal gyrus | 14 | -24 | -10 | 3.78 | <0.001 | 136 | n.s. |  |
| 19 | Right anterior cingulate cortex (ACC) | 12 | 30 | 24 | 3.78 | <0.001 | 112 | n.s. |  |
| 20 | Right middle cingulate cortex | 20 | -12 | 48 | 3.75 | <0.001 | 40 | n.s. |  |
| 21 | Left inferior frontal gyrus | -46 | 16 | 14 | 3.75 | <0.001 | 32 | n.s. |  |
| 22 | Right thalamus | 10 | -2 | -8 | 3.75 | <0.001 | 24 | n.s. |  |
| 23 | Right cerebellum | 26 | -46 | -52 | 3.72 | <0.001 | 40 | n.s. |  |
| 24 | Left precentral gyrus | -30 | -14 | 48 | 3.70 | <0.001 | 32 | n.s. |  |
| 25 | Left cerebellum | -6 | -52 | -26 | 3.69 | <0.001 | 40 | n.s. |  |
| 26 | SMA | -14 | -12 | 56 | 3.60 | <0.001 | 8 | n.s. |  |
| 27 | Right inferior parietal lobe | 52 | -44 | 54 | -4.91 | <0.001 | 2064 | <0.1 | 568 |
| 28 | Left inferior parietal lobe | -60 | -26 | 46 | -4.80 | <0.001 | 680 | n.s. |  |
| 29 | Right middle occipital lobe | 30  40 | -86  -82 | 30  24 | -4.54  -4.49 | <0.001  <0.001 | 1744 | n.s. |  |
| 30 | Left inferior parietal lobe | -48 | -52 | 52 | -4.18 | <0.001 | 616 | n.s. |  |
| 31 | Right postcentral gyrus | 30 | -42 | 74 | -4.07 | <0.001 | 304 | n.s. |  |
| 32 | Left middle cingulate cortex | -6  -6 | -34  -20 | 46  46 | -4.06  -3.87 | <0.001  <0.001 | 736 | n.s.  n.s. |  |
| 33 | Left precentral gyrus | -34 | -8 | 66 | -4.02 | <0.001 | 128 | n.s. |  |
| 34 | Left superior occipital lobe | -26 | -84 | 40 | -3.88 | <0.001 | 240 | n.s. |  |
| 35 | Right superior parietal lobe | 38 | -62 | 58 | -3.85 | <0.001 | 184 | n.s. |  |
| 36 | Left SMA | -10 | 2 | 76 | -3.77 | <0.001 | 8 | n.s. |  |
| 37 | Right calcarine sulcus | 22 | -62 | 12 | -3.76 | <0.001 | 24 | n.s. |  |
| 38 | Right paracentral lobe | 10 | -18 | 80 | -3.75 | <0.001 | 32 | n.s. |  |
| 39 | Left precuneus | -4 | -50 | 60 | -3.74 | <0.001 | 144 | n.s. |  |
| 40 | Right paracentral lobe | 10 | -36 | 54 | -3.74 | <0.001 | 120 | n.s. |  |
| 41 | Superior frontal gyrus | 2 | 34 | 58 | -3.72 | <0.001 | 32 | n.s. |  |
| 42 | Left paracentral lobe | -6 | -24 | 80 | -3.68 | <0.001 | 8 | n.s. |  |
| 43 | Left paracentral lobe | -8 | -22 | 80 | -3.66 | <0.001 | 16 | n.s. |  |
| 44 | Right SMA | 4 | 10 | 72 | -3.66 | <0.001 | 8 | n.s. |  |
| 45 | Left inferior frontal gyrus | -52 | 36 | 26 | -3.65 | <0.001 | 24 | n.s. |  |
| 46 | Left paracentral lobe | -4 | -22 | 78 | -3.65 | <0.001 | 8 | n.s. |  |
| 47 | Left SMA | -10 | 4 | 74 | -3.62 | <0.001 | 8 | n.s. |  |
| 48 | Right superior frontal gyrus | 18 | 72 | -4 | -3.60 | <0.001 | 8 | n.s. |  |

*SMA = supplementary motor area
